# Progress in Medicine

**Published:** 1898-10-15

**Authors:** 


### Progress in Medicine.

### DISEASES OF DIGESTIVE ORGANS.

(*Continued from page 443, Vol. XXIV.*)

**The various forms of Dyspepsia.**—Robin[20] refers to the fact that no symptoms may be presented by patients whose stomachs have lost all digestive power. The work may be entirely carried out by the intestine, and except perhaps for some constipation, there may be nothing to point to the state of affairs. A patient of his in good health except for psoriasis, was found to have neither hydrochloric acid, organic acids, or peptones in the contents of his stomach. W. Clifton Lyle[21] recommends the following procedure for testing the stomach contents. Congo paper is used for discovering whether free acid or acid salts only exist; a blue tint here shows the presence of acids. Boas' reagent indicates whether this is H.Cl.; if not, Uffelman's test is used for lactic acid. For pepsin, discs of hard-boiled egg are placed in tubes containing respectively water, H.Cl. solution, pepsin solution, and a solution containing H.Cl. and pepsin. These tests are simple, and easy of application, and to complete the diagnosis of cancer from other complaints he employs the iodide of potash test for rapidity of absorption, since the saliva contains the drug normally in five minutes after administration, when expectoration into a nitrate of silver solution gives a yellow colour. In cancer this reaction is delayed half an hour or more. Verhaegen[22] recognises four forms of dyspepsia, (*a*) simple chronic gastritis or catarrh, (*b*) gastritis with hyperchlorydria, (*c*) that with hypersecretion, and (*d*) neurotic dyspepsia. The first contains the common "flatulent, bilious, and atonic" forms with deficient motor power and acid, and excessive fermentations. For treatment he gives small meals of digestible food, with long intervals. Bitters may be given before meals, and H.Cl. after food. Lavage with warm water two or three times a week is also of use. (*b*) Hyperchlorydria is not shown by sour eructations, but by severe pain some hours after food relieved by alkalies. The diet should be milk and eggs only at first, with rest in bed, and very small quantities of bicarbonate of soda every half hour till the end of digestion. The H.Cl. may or may not be excessive, but the stomach is hypersensitive. (*c*) Hypersecretion is, he thinks, a later stage of (*b*) and the result of pyloric stenosis. The stomach is dilated, and even during fasting contains large amounts of acid secretions. Washing out the stomach with warm water containing an alkali, before the evening meal, moderates the secretion and destroys the fermentation. The diet should be albuminous. Roughly speaking, his divisions comprise hypo-acidity and hyperacidity, and the latter combined with the loss of motor power. Popoff[22] denies that gastritis is always accompanied by subacidity. In experimentally produced gastritis he found at first heightened digestive activity. He holds that there are two forms of inflammation—parenchymatous with increased secretion, as we see in gastric ulcer and often in alcoholism; and interstitial, where the glands are compressed, and subacidity finally results. Thus in carcinoma there is an absence of acid because the glands are constricted by the morbid tissue.

T. L. Chadbourne[24] records a number of experiments on digestive leucocytosis, showing that the phenomenon has no great diagnostic value. It is generally absent in carcinoma, but also not infrequently in other diseases and even in normal conditions of health, and in anæmias.

Hayem[25] enters on a crusade against the excessive use of alkalies and alkaline mineral waters as leading to over-secretion of H.Cl. in certain patients, *i.e.*, those whose glandular apparatus is active. Where the glands are atrophied, he thinks that alkalies cause depression and increase the dyspepsia. The question of the proper diet in hyperacidity has been much debated. Strauss and Aldor[26] recognise that the amount of carbohydrates must be reduced, that a purely nitrogenous diet is irritative, and recommend the abundant use of fats which diminish the formation of the total acids and of free H.Cl. From their experiments they assert that there is great toleration of fats in hyperchlorydria, and that the patient's nutrition is greatly improved by their use.

C. A. Aaron[27] speaks warmly of Einhorn's lamp for illuminating the stomach from inside, and gives rules for its use. The size and position of the stomach can be well made out. The respiratory movements of the stomach or their absence, and the presence of tumours are shown in many cases with ease.

Oppler,[28] while pointing out the uses of the method, denies that respiratory movements are sufficient for diagnosis between dilatation and gastroptosis of moderate degree. The movements of the intestinal tract have also been studied by the radioscope,[29] aided by capsules of celluloid filled with metallic bismuth, which the patient swallows.

C. A. Ewald,[30] of Berlin, writes upon the question, Should we drink with our meals? He says that the more fluid taken, the greater is the quantity of gastric juice secreted, and that the stomach accommodates itself to great variations in the amount taken either by absorption, or by the rapid passage of fluid into the intestine. Large quantities of liquid never remain long in the normal stomach; nor do moderate amounts of alcohol retard digestion according to Chittenden's researches. In healthy persons, then, moderate drinking at meals is a useful stimulant to digestion; but in dilatation no fluids should be given. In ulcer, irritating alcoholic drinks must be, of course, avoided; but otherwise, in stomach diseases, small amounts of fluid may be allowed with the meals.

Robin[31] recommends for some forms of fermentation draughts containing about one-fifth of a grain of fluoride of ammonium, and for butyric acid formation the double iodide of bismuth and cinchonidin after meals. Orexin has been employed by various authors for loss of appetite, while R. Frommel[32] has found it very efficacious in a few cases, where he tried it for the vomiting of pregnancy. A remarkable case of vomiting which occurred daily for eighteen years, in spite of treatment of various kinds, was cured by laparotomy. Mr. H. G. H. Maylon[33] opened the abdomen and then the stomach for supposed induration or stricture of the pylorus, but only found numerous adhesions to neighbouring organs. He separated all those along the lower border of the stomach, and, after healing had taken place, the patient was found to be cured. R. Tripier[34] recommends, for bleeding from the stomach, rectal injections of hot water of a temperature of 120 deg. three or four times a day, and oftener if the bleeding recurs. These are continued twice a day until the patient is completely restored to health, the ordinary treatment in other respects being employed. Gunprecht of Jena[35] has shown that it is possible to give food by hypodermic injections of oil or sugar, and thinks that when patient has to undergo a great strain, such as an operation, we may thus supply his needs with advantage. Paul Jacob reports that he kept a patient well nourished for three weeks by injections of olive oil under the skin, which was readily absorbed. A solution of more than 4 per cent. of sugar cannot be employed without irritation. W. Wingrave[36] gives a comparative analysis of the various starch ferments, such as malt extract and Taka diastase. He concludes that of the commercial ferments Taka is the most powerful and most reliable since it is most rapid in its action; that the organic acids so frequently present in the stomach do retard, but do not permanently kill the ferments, and when neutralised are harmless. Taka seems less influenced by them, and by tea, coffee, and alcohol than the others. H.Cl. and other mineral acids quickly destroy the ferments if in sufficient quantity. None of the ferments have any effect on uncooked starch.

R. S. Thompson[37] has shown specimens of sand excreted per rectum by a patient who had long suffered from gastric catarrh, with dilatation and neurotic symptoms, after being fed on milk and lime water with a little toast and fish. The sand, in quantities of half an ounce to an ounce, was passed with each motion for some months. It readily sank in water, and was brownish yellow in colour. Chemically it contained about 25 per cent. of organic matter and much pigment, possibly from the bile, and the remainder was composed of phosphate and carbonate of lime, with some calcic oxide. The passage of the sand seemed to cause no pain, and there was no mucus or blood in the motions.

**Abdominal Symptoms.**—Gross[38] discusses the difficulties of localising the source of abdominal pains. Those arising from the stomach radiate most commonly upwards and to the side, those from other organs are referred to the stomach or hypogastrium. Pain produced by pressure on Burckhart's points is often felt in the pit of the stomach, and that caused by pressure on the xiphoid process, is felt by many healthy persons. In hyperchlorydria the pain after meals rapidly relieved by alkalies or albumen, is not accompanied by the localised pain on pressure present generally in gastric ulcer, while that of hæmorrhagic erosions, violent as it is, is equally unaffected by pressure or change of position. In ileus at first, pain is not increased by pressure before peritonitis sets in. Pain arising from appendicitis is notoriously uncertain in its radiation, but like other types it may assist us as a guide to other and more weighty symptoms. G. W. Gray[39] lays down the following rules for calling in a surgeon in appendicitis: (*a*) It should be as early as possible in the fulminating, rapidly progressive form of the disease. (*b*) In moderately severe cases it should not be later than the third day. (*c*) In similar cases which constantly relapse before recovery has taken place, there is the greatest difficulty in deciding, and no definite rule can be given. (*d*) The recurrent attacks with intervals of health, if increasing in frequency, demand operation. The absence or presence of a tumour is no guide. F. Treves[40] mentions several classes of cases in which abdominal section has had unexpected results. The effect upon tuberculous peritonitis of laparotomy is well known. Aldibert has collected 308 cases in which opera-

ARMY MEDICAL LIBRARY

tion was followed by cure in 69·8 per cent. The most favourable results followed mere opening of the abdomen in general peritonitis without suppuration, and in the non-suppurative encysted forms. Again recovery has followed a similar operation in pylephlebitis, and even sarcomata have for a time retrograded and shrunk up for a time after laparotomy. A large class of cases in which recovery has followed laparotomy is of course the neurasthenic one, where no definite disease or adhesions have been found. Certain remarkable cures have undoubtedly resulted, but though in some cases dilatation of the stomach or colon, or other definite changes, have been shown, in others no physical cause of the symptoms could be traced.

[20] New York Med. Jour., April 9. [21] Georgia Med. Jour., June. [22] Lancet, Mar. 6. [23] Internat. Med. Mag., Feb. [24] Columbus Med. Jour, Mar. 15. [25] Lancet, May 28. [26] Brit. Med. Jour., Aug. 13. [27] New York Med. Jour., June 25. [28] Amer. Jour. Med. Sci., Jan. [29] Amer. Jour. Med. Sci., April. [30] Med. Rec., April 23. [31] Clin. Jour., July 6. [32] The Therapist, June 15. [33] New York Med. Jour., Feb. 19. [34] La Semaine Med., June 1. [35] La Semaine Med., April 20. [36] Lancet, May 7. [37] Glasgow Med. Jour., Mar. [38] New York Med. Jour., Feb. 12. [39] Charlotte Med. Jour., Feb. [40] Brit. Med. Jour., Mar. 5.

### DISEASES OF CHILDREN.

(*Continued from page* 10.)

**Pemphigus Neonatorum.**—Holt[9] reports a case of this affection which bears out the view of Strelitz and others, that certain forms of this disease are due to septic poisoning and not to syphilis. The infant was nine days old, and bore evidences of great neglect, although well nourished. There was no history of syphilis. Numerous bullæ were present, some of them containing pus, and there was also moderately severe purulent ophthalmia. From the contents of some of the bullæ pure cultures of the staphylococcus pyogenes albus were obtained. The patient died of exhaustion three days later, and at the necropsy punctate hæmorrhages were found in the suprarenals and the thymus, while the liver, spleen, and kidneys were intensely congested. Streptococci and staphylococci were present in the lungs, the spleen, the left kidney, and the liver.

**The Pseudo-membranous Anginas of Scarlatina.**—This important subject has been reviewed by Hirschfeld,[10] his conclusions being drawn from a careful study of 166 cases. His first group of 50 cases comprises the minor forms with slight angina, some isolated or confluent plaques on the tonsils, and slight enlargement of the submaxillary glands. The duration of this angina was from ten to fifteen days, no special treatment was required, and the patients all recovered. In the second group, which he designates the malignant form of scarlatinal angina, were 27 children, all of whom died. Most of the patients had been reduced in health by previous illness, such as measles, diphtheria, rickets, or syphilis. The angina was present at the outset or soon after, and the false membranes extended rapidly, the breath became fœtid, and sanious fluid came from the nostrils. The swelling of the ganglia was enormous, producing an extensive phlegmon of the neck, but not yielding pus on incision. Evidences of septicæmia were present in other parts of the body—*e.g.*, the eyes, the ears, the joints, and the lungs. Treatment seemed to be of no avail. In the third group, comprising 89 cases, the course of the angina was more chronic, and at first it had little effect on the scarlet fever; but an exacerbation or a recurrence was apt to follow with the development of malignant angina and septicæmia, and this usually preceded a fatal termination. In all of these groups the angina is due to the scarlatinal poison; while, in the second and third groups, the special virulence is due to a secondary streptococcic infection. As regards the treatment, in the third group of cases the author believes that injections of carbolic acid, 3 to 5 per cent. strength, into the tonsils were successful in 56 out of 60 cases.

**"Grimacing"—"Habit Chorea."**—This affection is found by Dr. Cheadle[11] to occur chiefly amongst the more neurotic children of the well-to-do, while "acute genuine or rheumatic chorea" is more common amongst the poor. Habit chorea is involuntary, reflex in origin, and is limited pretty closely to the age of from eight to twelve years. He finds that, as during the period of the first dentition twitchings and convulsions are not uncommon, so during the period of second dentition reflex disturbances are caused which manifest themselves in these grimaces. He has found that the affection usually reaches its height with the eruption of the eye teeth and late molars, and ceases when dentition is completed. Habit chorea is to be distinguished from true chorea in that the spasms are always limited to the muscles of the face, neck, and occasionally of one or other arm or shoulder; there is no derangement of the general health, and there is no association with rheumatism or other general disease. The most common of these involuntary movements are winking of the eyes, corrugations of the nose, sniffing and snorting of the nostrils, rapid and repeated elevations of the eyebrows, little shakes of the head, and shrugs of one or both shoulders. The treatment recommended is to maintain the general health at the highest possible level, and to secure to the patient freedom from undue excitement and from teasing, punishment, or rebuke on account of the grimaces. The parents may then be assured that, although the movements may persist or recur for months, they will eventually disappear when the second teething is fully completed.

[9] New York Med. Jour., Feb. 5, 1898; and Amer. Jour. Med. Sci., May, 1898. [10] Jahrb. f. Kinderheil., Bd. xliv., S. 237; and Amer. Jour. Med. Sci., May, 1898. [11] St. Mary's Hosp. Gaz., June, 1898.

